# The Hypodermic Syringe a Means of Diagnosis in Ovarian Disease

**Published:** 1870-10

**Authors:** Henry F. Walker

**Affiliations:** N. Y.


### The Hypodermic Syringe a Means of Diagnosis in Ovarian Disease.

BY HENRY F. WALKER, M. D., N. Y.

By aid of the microscope, with never so small an amount of the fluid contents of the tumor, a diagnosis can be made in every case. The means I would suggest, which possesses all the advantages of tapping with none of its hazards, is the hypodermic syringe, with the finest of needle points. This little instrument has often been used in diagnosticating purulent from serous effusions in the pleural cavity and pericardium, in detecting pus in cases of doubtful fluctuation under deep tissues, but has never to my knowledge been employed as a means of diagnosis in ovarian disease until within a few days, in the case herewith detailed.

The advantages it posssesses are these:—1st, efficiency; 2nd, harmlessness; 3rd, painlessness.

1st. Efficiency.—This is undoubted in determining the nature of the tumor, whether solid, cancerous, cancroid, or cystic. By plunging in the needle and retracting the piston sufficient fluid will be withdrawn by the suction exerted for microscopical diagnosis, even though it be of the most adhesive form of colloid growth. If it be proved a cyst with fluid contents, the kind of cyst may be demonstrated in many instances, for by introducing the needle at different parts of the abdomen, and comparing the character of the fluids withdrawn, it can readily be determined whether they be drawn from a single cyst with uniform contents, or from a multilocular tumor, containing fluids of various density and composition. This tells more than the clinical history, palpation, and all other means of diagnosis combined, for it lets us look within the tumor itself.

2nd. It is harmless.—The fine needle of the hypodermic syringe can be introduced even into an aneurism without danger, while the wound it makes in the sac of an ovarian cyst is so small that nature ignores it. The usual trocar makes a rent; this dissects its way between the tissues, and their contractility closes the wound.

3rd. The painlessness of the operation is of less importance to the surgeon than to the patient; but to the latter, to whom the preliminaries of examination are often more irksome than the grand operation itself, it is very desirable that diagnostic procedures should be painless as well as harmless.

